# Association of Increased CT-Attenuation of Visceral Adipose Tissue After Surgery with Poor Survival Outcomes in Patients with Stage II–III Gastric Cancer: A Retrospective Cohort Study

**DOI:** 10.3390/cancers17020235

**Published:** 2025-01-13

**Authors:** Sang Mi Lee, Geum Jong Song, Myoung Won Son, Jong Hyuk Yun, Moon-Soo Lee, Jeong Won Lee

**Affiliations:** 1 Department of Nuclear Medicine, Soonchunhyang University Cheonan Hospital, 31 Suncheonhyang 6-gil, Dongnam-gu, Cheonan 31151, Republic of Korea; 2 Department of Surgery, Soonchunhyang University Cheonan Hospital, 31 Suncheonhyang 6-gil, Dongnam-gu, Cheonan 31151, Republic of Korea

**Keywords:** computed tomography, gastric cancer, prognosis, recurrence, subcutaneous adipose tissue, visceral adipose tissue

## Abstract

Background/Objectives: This study aimed to investigate whether post-operative changes in the computed tomography (CT)-attenuation of subcutaneous (SAT) and visceral (VAT) adipose tissues were significantly associated with recurrence-free survival (RFS), peritoneal RFS, and overall survival (OS) in patients with stage II–III gastric cancer. Methods: This retrospective study analyzed 243 patients with stage II–III gastric cancer who underwent curative surgery. CT-attenuation values of SAT (SAT HU) and VAT (VAT HU) were measured from non-contrast-enhanced abdominopelvic CT images taken pre-operatively and 6 months post-operatively. Changes in SAT HU (ΔSAT HU) and VAT HU (ΔVAT HU) between the two CT scans were calculated. The prognostic value of these variables for predicting survival outcomes was assessed. Results: Correlation analyses showed that both ΔSAT HU and ΔVAT HU were significantly positively correlated with T stage, TNM stage, and tumor size (*p* < 0.05). In the multivariate survival analysis, ΔVAT HU emerged as an independent significant predictor for RFS (*p* = 0.002, hazard ratio, 2.437), peritoneal RFS (*p* = 0.023, hazard ratio, 2.457), and OS (*p* = 0.043, hazard ratio, 2.204) after adjusting for age, sex, histopathological classification, T stage, and N stage. Patients with high ΔVAT HU had worse RFS, peritoneal RFS, and OS compared to those with low ΔVAT HU. Conclusions: Change in CT-attenuation of VAT following surgery was significantly correlated with tumor characteristics and was a significant predictor of RFS, peritoneal RFS, and OS in patients with stage II–III gastric cancer.

## Simple Summary

The clinical significance of changes in computed tomography (CT)-attenuation after surgery has not yet been reported in gastric cancer patients. In this study, we measured the CT-attenuation of both subcutaneous (SAT) and visceral (VAT) adipose tissue in pre-operative and 6-month post-operative CT scans of stage II–III gastric cancer patients undergoing curative surgery, and investigated whether changes in CT-attenuation of SAT (ΔSAT HU) and VAT (ΔVAT HU) are associated with survival outcomes. Our study demonstrated that patients with tumors in the upper stomach and advanced tumor stages exhibited higher values of both ΔVAT HU and ΔSAT HU compared to others. Furthermore, ΔVAT HU was an independent risk factor for cancer recurrence, particularly peritoneal recurrence, and mortality on the multivariate survival analysis. Our study suggests that a change in the CT-attenuation of VAT after surgery might serve as a potential imaging biomarker for predicting cancer recurrence and overall survival in locally advanced gastric cancer patients.

## 1. Introduction

Gastric cancer ranks fifth worldwide in both incidence and mortality [1]. In patients with advanced gastric cancer without distant metastasis, the standard curative treatment recommended is radical gastrectomy and regional lymph node dissection followed by adjuvant chemotherapy [2]. Nevertheless, approximately 30% of patients treated with curative surgery for advanced gastric cancer suffer a recurrence, with 5-year overall survival (OS) rates ranging from 12.3 to 59.3% for stage II–III patients [3,4]. The peritoneum is the most frequent site of recurrence in gastric cancer, accounting for up to 58.8% of cases after curative surgery [3,5]. Moreover, peritoneal recurrence is distinguished by a particularly poor prognosis, with median survival times ranging only between 5.0 and 15.0 months [6,7]. Consequently, numerous studies have been conducted to identify risk factors for predicting peritoneal recurrence following surgical resection [7,8,9]. Earlier research identified the depth of tumor invasion, the number of metastatic lymph nodes, and the diffuse/mixed histopathological type gastric cancer as significant predictive tumor factors of peritoneal metastasis [3,7,8].

Recently, it has been suggested that, in addition to tumor factors, the condition of the microenvironment in visceral adipose tissue (VAT) is a key factor for the peritoneal recurrence of gastric cancer [10,11]. Studies have found that gastric cancer cells interact with adipocytes in the VAT, resulting in adipocyte dedifferentiation and inflammatory conditions with fibrosis, and subsequently, these dedifferentiated adipocytes promote the growth and peritoneal metastasis of gastric cancer cells [10,11,12]. Recent studies have shown a significant positive correlation between the degree of inflammatory and fibrotic changes in VAT and its computed tomography (CT)-attenuation, expressed as mean Hounsfield units (HU), on non-contrast-enhanced CT images [13,14]. Therefore, CT-attenuation of VAT is proposed as an imaging parameter that can reflect the qualitative characteristics of the VAT microenvironment, and an increased mean HU of VAT is significantly associated with worse survival outcomes for patients with various cancers in the abdominopelvic cavity, including gastric cancer [9,14,15,16].

In gastric cancer patients who underwent curative resection, both VAT quantity and body weight significantly decreased within the first 6–12 months following the surgery [17,18]. Since the loss of VAT quantity after surgery indicates nutritional deficiency and a negative energy balance, a marked reduction in VAT significantly correlated with shorter recurrence-free survival (RFS) and OS in patients with gastric cancer [17,19]. In addition to VAT quantity, qualitative changes in VAT might be altered after surgery for gastric cancer. Considering the significant relationship between the VAT microenvironment and the progression of the disease, it is hypothesized that changes in HU of VAT between pre-operative and post-operative CT images could have a significant association with survival outcomes of gastric cancer, potentially allowing the prediction of prognosis using imaging parameters measured from routine imaging examinations. However, the clinical importance of changes in adipose tissue CT-attenuation after surgery has not yet been reported in gastric cancer patients.

Hence, in this study, we measured CT-attenuation of both subcutaneous adipose tissue (SAT) and VAT in pre-operative and 6-month post-operative CT scans of stage II–III gastric cancer patients undergoing radical gastrectomy, and investigated whether changes in the CT-attenuation of SAT and VAT are significantly associated with RFS, peritoneal RFS, and OS.

## 2. Materials and Methods

### 2.1. Patients

The electronic medical records of patients histopathologically confirmed with gastric cancer who underwent surgical resection at Soonchunhyang University Cheonan Hospital from January 2011 to December 2021 were retrospectively reviewed. Of these patients, we included patients who (1) were clinically M0 stage on staging imaging studies, (2) underwent radical curative surgery, (3) were histopathologically diagnosed with stage II–III gastric cancer on surgical specimen evaluation, and (4) underwent both pre-operative (CT1) and 6-month post-operative (CT2) abdominopelvic CT scans. We excluded patients who (1) had a prior history of another malignancy or major abdominal surgery, (2) were diagnosed with peritoneal metastases on surgical findings or revealed malignant cells on peritoneal washing cytology, (3) showed peritoneal recurrence on CT2 images, and (4) had inadequate CT images for measuring CT-attenuation of SAT and VAT. A total of 243 patients with stage II–III gastric cancer were finally included in this study based on the inclusion and exclusion criteria.

All patients underwent pre-operative examinations including blood tests, gastroduodenoscopy, and abdominopelvic CT. Radical gastrectomy with D2 lymphadenectomy followed the staging work-up. The median interval between the pre-operative CT scan and surgery was 17.0 days (range, 2–48 days). Adjuvant chemotherapy was recommended for all patients. A 6-month post-operative abdominopelvic CT scan was conducted for surveillance following the surgery, with a median interval of 6.6 months (range, 5.2–10.1 months). Regular clinical follow-ups after treatment were conducted using gastroduodenoscopy and abdominopelvic CT.

### 2.2. Measurements of CT-Attenuation of Adipose Tissue

**Figure 1:**
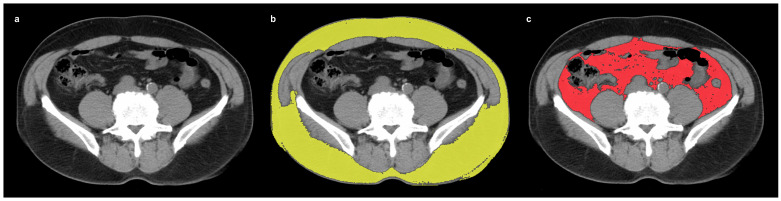
An example of measuring the CT-attenuation of SAT and VAT. On a transaxial CT image at the level of the L4–L5 vertebrae (**a**), SAT and VAT areas were automatically selected using a CT-attenuation threshold. SAT (**b**) was defined as extra-peritoneal adipose tissue with a CT-attenuation threshold between −190 and −30 HU (yellow area). VAT (**c**) was defined as the intra-abdominal adipose tissue between a CT-attenuation threshold of −150 and −50 HU (red area). (CT, computed tomography; HU, Hounsfield units; SAT, subcutaneous adipose tissue; VAT, visceral adipose tissue).

Non-contrast-enhanced CT images from CT1 and CT2 (GoldSeal CT750, GE Healthcare, Chicago, IL, USA) were retrospectively reviewed by a single reviewer (J.W.L.), blinded to the patients’ clinical outcomes. CT-attenuation of SAT and VAT was measured using OsiriX version 14.0.1 software (Pixmeo SARL, Geneva, Switzerland). Since the vast majority of previous studies have used non-contrast-enhanced CT images to measure the CT-attenuation of SAT and VAT, we also used the same method [9,13,14,15]. A single transaxial CT image at the L4–L5 vertebrae level was selected to minimize the potential influence of the primary gastric cancer lesion [9]. The SAT and VAT areas were automatically identified in the transaxial CT image. The SAT was defined as extra-peritoneal adipose tissue with a CT-attenuation range between −190 and −30 HU, and VAT was defined as intra-abdominal adipose tissue with a CT-attenuation range between −150 and −50 HU (Figure 1) [20,21]. Mean CT-attenuation values for SAT (SAT HU) and VAT (VAT HU) were then measured from areas on CT1 and CT2. The percent changes in mean CT-attenuation for SAT (ΔSAT HU) and VAT (ΔVAT HU) between CT1 and CT2 were calculated as follows: (ΔCT-attenuation) = [(mean CT-attenuation on CT1) − (mean CT-attenuation on CT2)]/(mean CT-attenuation on CT1) × 100 (%).

### 2.3. Statistical Analysis

Pairwise comparisons between ΔSAT HU and ΔVAT HU were conducted using the Wilcoxon signed-rank test. The association between adipose HU parameters and clinicopathological factors was assessed using the Kruskal–Wallis test and Mann–Whitney U test. For parameters that achieved statistical significance in the Kruskal–Wallis test, post hoc comparisons using Dunne’s test were conducted. Pearson’s correlation coefficients were used to evaluate the relationship between adipose HU parameters and tumor size. The prognostic value of SAT HU on CT1, VAT HU on CT1, SAT HU on CT2, VAT HU on CT2, ΔSAT HU, and ΔVAT HU in predicting RFS, peritoneal RFS, and OS was examined using univariate and multivariate Cox proportional hazard regression analyses. The survival time was defined as the interval from the day of surgery to the day of cancer recurrence, peritoneal recurrence, death, or the last follow-up visit. Peritoneal recurrence was characterized as findings of peritoneal seeding nodules, increased peritoneal density, abnormally thickened intestinal walls, peribiliary infiltration, massive ascites, or a Krukenberg tumor on imaging examinations or the presence of cancer cells in ascites or peritoneal tissue. For survival analysis, all adipose tissue HU parameters were dichotomized based on optimal cut-off values determined by receiver operating characteristic (ROC) curve analysis. Only the adipose tissue HU parameters that demonstrated statistical significance in the univariate survival analysis were included in the multivariate survival model, adjusted for age, sex, histopathological classification, T stage, and N stage. Cumulative RFS, peritoneal RFS, and OS curves were estimated using the Kaplan–Meier method, and differences between the survival curves were evaluated using a log-rank test. All statistical analyses were performed using MedCalc Statistical Software version 23.0.2 (MedCalc Software Ltd., Ostend, Belgium). A *p*-value of <0.05 was deemed statistically significant.

## 3. Results

### 3.1. Patient Characteristics

The clinicopathological characteristics of the 243 enrolled patients are summarized in Table 1. Among these patients, 155 (63.8%) had primary tumor lesions in the lower-third portion of the stomach, and the most common histopathology was the poorly cohesive type (60.9%). Histopathological assessment revealed that 175 patients (72.0%) had lymph node metastasis, and 117 patients (48.1%) were diagnosed with stage III gastric cancer. All patients had negative surgical margins on histopathological evaluation. After surgery, 240 patients (98.8%) received adjuvant chemotherapy, and all of these patients had completed chemotherapy. At 6-month post-operative CT images, 209 patients (86.0%) and 219 patients (90.1%) exhibited increases in ΔSAT HU and ΔVAT HU, respectively. The ΔVAT HU values were significantly higher than the ΔSAT HU values in the pairwise comparison (*p* = 0.005).

### 3.2. Correlation Analysis

The results of the correlation analysis between adipose tissue HU parameters and clinicopathological factors are presented in Table 2. For SAT HU on CT1, men, and patients who were ≥60 years had significantly higher values than women and those who were <60 years, respectively (*p* < 0.05), whereas no significant relationship was observed between SAT HU on CT1 and tumor factors. VAT HU on CT1 was significantly associated with the T stage (*p* = 0.047), showing higher values in patients with the T4 stage compared to those with the T2 stage on post hoc analysis (*p* < 0.05). Patients with TNM stage III also tended to have higher VAT HU values on the CT1 than those with stage II, albeit with borderline statistical significance (*p* = 0.063). SAT HU on CT2, VAT HU on CT2, ΔSAT HU, and ΔVAT HU parameters showed significant associations with tumor location, surgery type, T stage, and TNM stage (*p* < 0.05 for all). In the post hoc analysis, tumors in the upper third portion of the stomach had significantly higher values of SAT HU on CT2, ΔSAT HU, and ΔVAT HU compared to those in the middle and lower portions (*p* < 0.05), and higher values of VAT HU on CT2 compared to those in the lower portion (*p* < 0.05). Regarding surgery type, patients who underwent total gastrectomy had significantly higher values of SAT HU on CT2, VAT HU on CT2, ΔSAT HU and ΔVAT HU compared to those who underwent subtotal gastrectomy (*p* < 0.05). In terms of T stage, patients with T3 and T4 had significantly higher values of SAT HU on CT2, VAT HU on CT2, ΔSAT HU and ΔVAT HU compared to those with T2 (*p* < 0.05). Additionally, patients with TNM stage III exhibited significantly higher values of SAT HU on CT2 (*p* < 0.001), VAT HU on CT2 (*p* < 0.001), ΔSAT HU (*p* = 0.001) and ΔVAT HU (*p* = 0.014) compared to those with stage II.

The correlation analysis with tumor size, SAT HU on CT2 (*p* = 0.009; correlation coefficient, 0.167; 95% CI, 0.042–0.287), VAT HU on CT2 (*p* = 0.010; correlation coefficient, 0.165; 95% CI, 0.040–0.285), ΔSAT HU (*p* = 0.002; correlation coefficient, 0.197; 95% CI, 0.073–0.315), and ΔVAT HU (*p* = 0.001; correlation coefficient, 0.206; 95% CI, 0.083–0.324) demonstrated significant yet weak positive correlations with tumor size, while neither SAT HU (*p* = 0.918) nor VAT HU (*p* = 0.977) on CT1 exhibited significant correlation.

### 3.3. Survival Analysis

The median follow-up duration of the patients was 60.9 months (range, 6.9–162.5 months). At the time of analysis, 73 patients (30.0%) experienced cancer recurrence after surgery, and 41 patients (16.9%) had died. Of those with recurrence, peritoneal recurrence occurred in 43 patients (17.7%). None of the patients experienced a recurrence by the 6-month post-operative CT scan. The 5-year RFS, peritoneal RFS, and OS rates were 70.2% [95% confidence interval (CI), 64.3–76.1%], 82.3% (95% CI, 77.3–87.3%), and 85.2% (95% CI, 80.5–89.9%), respectively.

Before assessing the prognostic values of SAT and VAT HU parameters for predicting RFS, peritoneal RFS, and OS, all adipose tissue CT-attenuation parameters were categorized into two groups based on the optimal cut-off values determined by ROC curve analysis. The cut-off values were −97.2 HU for SAT HU on CT1, −82.9 HU for VAT HU on CT1, −82.3 HU for SAT HU on CT2, −70.0 HU for VAT HU on CT2, 9.3% for ΔSAT HU, and 20.3% for ΔVAT HU.

In the univariate survival analysis, VAT HU on CT1, SAT HU on CT2, VAT HU on CT2, ΔSAT HU, and ΔVAT HU were significantly associated with RFS, peritoneal RFS, and OS (*p* < 0.05; Table 3). Conversely, SAT HU on CT1 was not significantly associated with any survival outcomes (*p* > 0.05, Table 3).

**Figure 2:**
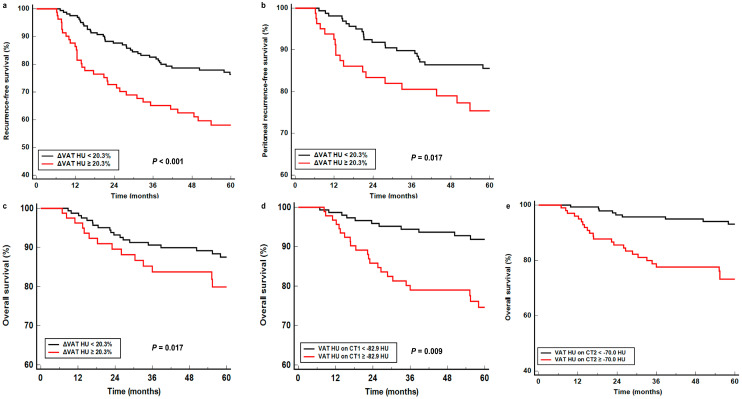
Cumulative recurrence-free survival (**a**) and peritoneal recurrence-free survival (**b**) curves according to ΔVAT HU. Cumulative overall survival curves based on ΔVAT HU (**c**), VAT HU on CT1 (**d**), and VAT HU on CT2 (**e**). (CT1, pre-operative computed tomography; CT2, 6-month post-operative computed tomography; HU, Hounsfield unit; VAT, visceral adipose tissue; ΔVAT HU, percent changes in mean computed tomography-attenuation for visceral adipose tissue between pre-operative and 6-month post-operative scans).

VAT HU on CT1, SAT HU on CT2, VAT HU on CT2, ΔSAT HU, and ΔVAT HU, having shown statistical significance in univariate analysis, were included in multivariate survival analysis for RFS, peritoneal RFS, and OS along with age, sex, histopathological classification, T stage, and N stage as covariates (Table 4). The results from the multivariate survival analysis revealed that ΔVAT HU remained a significant predictor for RFS (*p* = 0.002; hazard ratio, 2.437), peritoneal RFS (*p* = 0.023; hazard ratio, 2.457), and OS (*p* = 0.043; hazard ratio, 2.204). Patients with high ΔVAT HU experienced significantly worse RFS, peritoneal RFS, and OS than those with low ΔVAT HU (Figure 2a–c). The 5-year RFS, peritoneal RFS, and OS rates for patients with ΔVAT HU < 20.3% were 76.3% (95% CI, 69.6–83.0%), 85.6% (95% CI, 80.0–91.2%), and 87.5% (95% CI, 82.2–92.8%), while for those with ΔVAT HU ≥ 20.3%, the rates were 58.1% (95% CI, 47.1–69.1%), 75.0% (95% CI, 65.0–85.1%), and 79.9% (95% CI, 70.3–89.5%), respectively. In addition to ΔVAT HU, VAT HUs on both CT1 and CT2 were independent predictors for OS (*p* = 0.049; hazard ratio, 2.016 for VAT HU on CT1; *p* = 0.009; hazard ratio, 2.515 for VAT HU on CT2), and showed borderline statistical significance for peritoneal RFS (*p* = 0.072 for VAT HU on CT1 and *p* = 0.066 for VAT HU on CT2). VAT HU on CT2 also revealed a borderline statistical significance for predicting RFS (*p* = 0.053). Patients with VAT HU on CT1 ≥ −82.9 HU and on CT2 ≥ −70.0 HU exhibited poorer OS compared to those with VAT HU on CT1 < −82.9 HU and on CT2 < −70.0 HU (Figure 2d,e). On the other hand, SAT HU on CT2 and ΔSAT HU did not demonstrate statistical significance for predicting RFS, peritoneal RFS, and OS (*p* > 0.05 for all).

## 4. Discussion

Gastric cancer is one of the malignancies that grows in an adipose tissue-dominated microenvironment, which inevitably leads to close contact between gastric cancer cells and adipocytes in VAT [9,10,11]. The cross-talk between the gastric cancer cells and adipocytes results in the dedifferentiation of adipocytes, characterized by a reduced number and size of lipid droplets and diminished expression of adipocyte markers, alongside increased gene expression levels of fibroblast-specific protein 1 and various inflammatory cytokines, including interleukin-6 (IL-6) [11,22]. This dedifferentiation promotes the phenotypic conversion of adipocytes to cancer-associated fibroblasts [11,23]. In turn, cancer-associated fibroblasts supply gastric cancer cells with free fatty acids, which fuel cell metabolism, and enhance tumor growth and peritoneal metastasis [23,24]. Additionally, by secreting various adipokines, cancer-associated fibroblasts contribute to the downregulation of CD4+ memory T-cells and CD8+ cytotoxic T-cells, and boost the recruitment of M2 macrophages and fibrosis in the adipose tissue [10,11,25]. These inflammatory and fibrotic modifications in the adipose tissue create a tumor microenvironment conducive to cancer cell survival and progression [11,23,25]. In a previous study involving non-human primates, increased CT-attenuation of VAT was correlated with smaller adipocytes with lower intracellular lipid content and increased extracellular matrix accumulation, which are also evident in the adipose tissue microenvironment with cancer-associated fibroblasts [9,13]. In another study that analyzed surgical specimens from colorectal cancer patients, the pre-operative CT showed that VAT HU was significantly positively correlated with the degree of IL-6 expression in the VAT [14]. Consequently, VAT HU is proposed as an imaging parameter that can indicate qualitative characteristics of VAT [9,15,26]. In clinical studies, the increased CT-attenuation of VAT on pre-treatment CT images consistently exhibited significant association with poor survival outcomes in various kinds of cancers, including colorectal cancer, renal cell carcinoma, and hepatocellular carcinoma [16,27,28]. In studies of gastric cancer, VAT HU values from the pre-operative CT scan could predict occult peritoneal metastases [29]. Furthermore, in recent two studies with gastric cancer patients who underwent radical gastrectomy, increased VAT HU on pre-operative CT images was also significantly linked with higher risk of peritoneal recurrence and mortality [9,26]. In the current study, VAT HUs on both pre-operative and post-operative CT scans independently predicted OS in multivariate survival analysis, in line with other studies.

In addition to VAT HU, previous studies have found the prognostic significance of SAT HU for predicting survival in malignant diseases [30,31,32,33]. In a similar manner to VAT, cancer cells induce dedifferentiation and reprogramming of adipocytes in SAT, and during this process, adipocytes secrete adipokines that can enhance systemic inflammatory reactions [30,33]. Moreover, among patients with cancer cachexia, browning of SAT with increased vascularity can be observed as an adjustment to energy expenditure [30]. All of these changes lead to an increase in SAT HU; thereby, SAT HU could become significantly associated with survival [30,31,33]. A recent study with gastric cancer patients treated with surgery found that high SAT HU on pre-operative CT images was associated with poor OS [30]. In the presented study, we measured SAT HU as well as VAT HU from CT1 and CT2 images, and in univariate survival analysis, SAT HU on CT2 was significantly associated with RFS, peritoneal RFS, and OS. However, in multivariate survival analysis, it failed to show statistical significance for predicting survival outcomes, suggesting that parameters of VAT CT-attenuation might have more significant prognostic implications than those of SAT CT-attenuation.

In this study, along with VAT HU and SAT HU on CT1 and CT2, we measured changes in VAT HU and SAT HU between pre-operative and 6-month post-operative CT scans in gastric cancer patients. Among the patients enrolled in our study, 86.0% and 90.1% demonstrated ΔSAT HU > 0% and ΔVAT HU > 0%, respectively, indicating enhanced CT-attenuation of both SAT and VAT in the majority of gastric cancer patients undergoing curative surgery. Furthermore, patients with tumors in the upper stomach and advanced tumor stages exhibited significantly higher values of both ΔVAT HU and ΔSAT HU compared to others. Cancers in the upper third of the stomach are known to possess distinct biological characteristics, presenting a higher risk of both lymphatic and hematogenous tumor spread and poorer survival than those in the middle and distal thirds of the stomach [34]. Therefore, our findings suggested that qualitative changes in SAT and VAT were more pronounced after surgery in patients with advanced-stage and aggressive gastric cancers. Given that patients undergoing total gastrectomy also exhibited higher SAT HU on CT2, VAT HU on CT2, ΔVAT HU, and ΔSAT HU than those receiving subtotal gastrectomy, it implies that post-operative changes from more extensive surgeries may influence the increase in VAT HU and SAT HU. Furthermore, in a previous study, types of reconstruction procedures after gastrectomy also affected the condition of VAT [35]. On the other hand, total gastrectomy is commonly performed on larger, more aggressive cancers or tumors in the upper third of the stomach [36]. Moreover, we measured VAT HU and SAT HU at the level of the L4–L5 vertebrae to mitigate the potential effects of primary gastric cancer and post-operative changes. Consequently, the observed increase in CT-attenuation of VAT and SAT after surgery is likely associated with tumor characteristics, rather than solely attributable to surgical modifications.

In addition to a significant relationship between tumor aggressiveness and changes in SAT and VAT HU, our study also found that ΔVAT HU was significantly associated with RFS, peritoneal RFS, and OS in gastric cancer patients according to the multivariate survival analysis, whereas ΔSAT was not. Patients with ΔVAT HU ≥ 20.3% exhibited significantly poorer survival outcomes compared to those with ΔVAT HU < 20.3%. Since this is the first study to report the prognostic significance of ΔVAT HU in gastric cancer patients, the reason for this association remains unclear. However, two potential explanations exist: the CT-attenuation of adipose tissue is known to be positively associated with adipose tissue microenvironment, but negatively with nutritional status [9,30]. Therefore, increased VAT HU on the 6-month post-operative CT scan might indicate enhanced inflammatory changes in VAT with poor nutrition after surgery, which gradually leads to a clinical condition that favors cancer recurrence and peritoneal metastasis [9,17,23,25,26]. Another possible explanation is the presence of occult metastatic gastric cancer cells in the peritoneum. Although we excluded patients diagnosed with peritoneal metastases during surgery and revealed findings of peritoneal recurrence on their 6-month post-operative CT scan, occult peritoneal metastasis might still have been present at the time of post-operative CT scanning. This might potentially contribute to increased VAT HU on CT2 and ΔVAT HU and adverse survival outcomes [9,29]. Due to poor vascularization and dense collagen deposition in peritoneal metastatic lesions, the efficacy of conventional systemic chemotherapy for treating peritoneal metastasis has been reported to be limited [10,37]. Consequently, multiple clinical trials have been conducted to prevent peritoneal recurrence by implementing prophylactic treatments, such as intraperitoneal chemotherapy, in gastric cancer patients at high risk for peritoneal metastasis [37,38]. Since our study revealed that patients with locally advanced gastric cancer, those who exhibited increased ΔVAT HU after surgery faced a higher risk of cancer recurrence, particularly peritoneal recurrence; hence, rigorous monitoring for cancer recurrence during follow-up is essential for these patients. Furthermore, these patients could be prime candidates for trials of prophylactic therapy targeting peritoneal recurrence.

The present study has several limitations: first, the patient sample was retrospectively selected from a single medical center, necessitating further multi-center studies with larger patient populations to validate our results; second, owing to the retrospective design of the study, the interval between surgery and post-operative CT scans varied, potentially impacting the results; third, peritoneal recurrence was primarily diagnosed using imaging examinations, which might lead to misclassification of recurrence sites; fourth, post-operative complications are notable factors that negatively affect survival outcomes [39,40]. However, due to the retrospective nature of this study, data regarding post-operative complications were not available. Therefore, the impact of post-operative complications on the prognostic significance of ΔVAT HU should be further investigated. Finally, the underlying mechanism of the relationship between ΔVAT HU and survival outcomes in gastric cancer warrants additional investigation through histopathological evaluation.

## 5. Conclusions

In stage II–III gastric cancer patients treated with curative surgery, ΔVAT HU was a significant independent prognostic factor for predicting RFS, peritoneal RFS, and OS. Both ΔVAT HU and ΔSAT HU significantly correlated with tumor location, size, and stage, and patients with high ΔVAT HU experienced worse survival outcomes than those with low ΔVAT HU. The change in CT-attenuation of VAT after surgery might serve as a potential imaging biomarker for predicting cancer recurrence, especially peritoneal recurrence, in patients with locally advanced gastric cancer.

## Figures and Tables

**Table 1:** Patient characteristics (*n* = 243).

Characteristics		Number of Patients (%)	Median (Range)
Age (years)			60 (33–88)
Sex	Men	174 (71.6%)	
	Women	69 (28.4%)	
Histopathological classification	Papillary/tubular type	75 (30.9%)	
	Poorly cohesive type	148 (60.9%)	
	Mucinous	12 (4.9%)	
	Others	8 (3.3%)	
Lauren classification	Intestinal type	72 (29.6%)	
	Diffuse/indeterminate type	171 (70.4%)	
Tumor location	Upper	59 (24.3%)	
	Middle	29 (11.9%)	
	Lower	155 (63.8%)	
Tumor size (cm)			4.5 (0.7–18.0)
Surgery type	Subtotal gastrectomy	171 (70.4%)	
	Total gastrectomy	69 (28.4%)	
	Proximal gastrectomy	3 (1.2%)	
Pathologic T stage	T2	36 (14.8%)	
	T3	134 (55.1%)	
	T4	73 (30.0%)	
Pathologic N stage	N0	68 (28.0%)	
	N1	55 (22.6%)	
	N2	47 (19.3%)	
	N3	73 (30.0%)	
TNM stage	Stage II	126 (51.9%)	
	Stage III	117 (48.1%)	
Adjuvant chemotherapy	Yes	240 (98.8%)	
	No	3 (1.2%)	
SAT HU on CT1 (HU)			−98.22 (−117.41–−58.90)
VAT HU on CT1 (HU)			−86.77 (−109.76–−53.86)
SAT HU on CT2 (HU)			−85.07 (−106.21–−41.55)
VAT HU on CT2 (HU)			−75.29 (−100.66–−54.68)
ΔSAT HU (%)			12.4 (−22.8–65.0)
ΔVAT HU (%)			14.7 (−34.3–48.9)

CT1, pre-operative computed tomography; CT2, 6-month post-operative computed tomography; HU, Hounsfield unit; SAT, subcutaneous adipose tissue; VAT, visceral adipose tissue; ΔSAT HU, percent changes in mean computed tomography-attenuation for subcutaneous adipose tissue between pre-operative and 6-month post-operative scans; ΔVAT HU, percent changes in mean computed tomography-attenuation for visceral adipose tissue between pre-operative and 6-month post-operative scans.

**Table 2:** Correlation analyses of SAT and VAT HU parameters with clinicopathological factors.

Clinicopathologial Factors		SAT HU on CT1 (HU)	VAT HU on CT1 (HU)	SAT HU on CT2 (HU)	VAT HU on CT2 (HU)	ΔSAT HU (%)	ΔVAT HU (%)
Age	≥60 years	−97.4 (−102.0–−90.1)	−86.9 (−93.2–−78.8)	−86.1 (−94.2–−74.6)	−72.3 (−78.2–−66.8)	11.1 (4.1–22.1)	15.0 (8.0–21.4)
	<60 years	−98.8 (−103.8–−94.8)	−86.6 (−93.9–−79.7)	−71.4 (−78.7–−64.6)	−71.4 (−78.7–−64.6)	12.6 (5.0–21.1)	14.3 (8.2–22.4)
	*p*-value	0.018	0.687	<0.001	0.641	0.572	0.840
Sex	Men	−96.5 (−101.4–−90.2)	−86.4 (−93.2–−78.5)	−80.3 (−88.4–−70.1)	−74.2 (−78.4–−68.7)	13.2 (4.8–22.6)	14.6 (8.3–22.0)
	Women	−103.1 (−105.7–−98.8)	−88.4 (−94.3–−80.7)	−91.8 (−98.0–−79.6)	−70.3 (−78.4–−65.2)	11.6 (4.5–19.4)	15.1 (8.0–22.8)
	*p*-value	<0.001	0.173	0.001	0.131	0.666	0.973
Histopathological classification	Papillary/tubular type	−97.6 (−101.8–−91.4)	−86.1 (−92.5–−78.8)	−84.3 (−94.2–−75.8)	−73.7 (−80.0–−66.3)	9.5 (4.3–18.7)	12.3 (6.6–21.4)
	Poorly cohesive type	−101.8 (−107.3–−94.7)	−95.4 (−98.2–−85.1)	−95.3 (−98.8–−79.5)	−80.6 (−84.9–−67.7)	13.7 (4.6–22.5)	16.2 (8.9–22.6)
	Mucinous	−98.5 (−103.2–−92.5)	−86.7 (−93.2–−79.4)	−85.3 (−94.4–−72.8)	−71.4 (−77.7–−65.7)	8.1 (3.6–10.6)	12.1 (9.7–16.7)
	*p*-value	0.114	0.099	0.353	0.231	0.209	0.276
Lauren classification	Intestinal type	−97.1 (−101.8–−90.0)	−85.9 (−93.0–−78.1)	−84.9 (−93.5–−76.2)	−71.6 (−77.9–−66.1)	9.8 (5.0–20.8)	14.1 (6.7–22.4)
	Diffuse/indeterminate type	−98.7 (−103.3–−92.8)	−86.9 (−93.3–−79.5)	−80.1 (−88.5–−75.6)	−72.1 (−78.5–−65.6)	13.2 (4.6–22.5)	15.1 (9.2–22.0)
	*p*-value	0.111	0.672	0.070	0.935	0.273	0.384
Tumor location	Upper	−98.5 (−103.3–−93.3)	−89.0 (−94.0–−79.0)	−77.1 (−85.7–−63.6)	−67.0 (−74.0–−63.3)	20.0 (13.4–32.2)	22.4 (16.3–25.9)
	Middle	−99.5 (−103.4–−95.2)	−86.2 (−93.2–−80.8)	−86.9 (−95.5–−73.5)	−71.5 (−77.1–−68.3)	15.1 (6.4–21.3)	16.1 (9.9–21.1)
	Lower	−97.6 (−102.4–−91.4)	−86.6 (−93.2–−78.7)	−87.9 (−96.2–−78.1)	−74.3 (−80.9–−67.0)	8.1 (2.6–7.5)	11.9 (6.3–18.4)
	*p*-value	0.178	0.544	<0.001	<0.001	<0.001	<0.001
Surgery type	Total gastrectomy	−98.0 (−103.1–−92.0)	−87.3 (−93.3–−80.7)	−76.8 (−86.0–−68.8)	−67.5 (−74.0–−61.4)	20.0 (13.4–30.9)	20.8 (14.4–25.6)
	Subtotal gastrectomy	−98.2 (−102.9–−93.1)	−86.6 (−93.4–−79.2)	−88.3 (−96.3–−78.4)	−74.3 (−80.4–−67.1)	8.3 (2.8–17.5)	12.2 (6.3–18.7)
	Proximal gastrectomy	−103.3 (−104.1–−91.0)	−90.3 (−93.2–−74.0)	−87.2 (−89.5–−84.6)	−71.2 (−72.3–−68.5)	13.5 (6.1–15.1)	21.2 (6.4–22.4)
	*p*-value	0.856	0.879	<0.001	<0.001	<0.001	<0.001
T stage	T2	−98.9 (−103.8–−95.4)	−90.3 (−94.9–−83.4)	−93.7 (−96.0–−82.8)	−77.2 (−83.3–−71.2)	7.5 (1.3–14.5)	6.9 (1.1–9.3)
	T3	−97.5 (−102.8–−91.7)	−86.6 (−93.3–−80.1)	−84.4 (−93.9–−73.2)	−71.4 (−77.7–−65.2)	12.0 (4.6–22.5)	14.9 (82.2–22.1)
	T4	−98.9 (−103.2–−91.9)	−84.9 (−92.5–−76.6)	−82.4 (−93.4–−68.1)	−70.0 (−77.8–−64.4)	15.1 (5.6–24.8)	14.4 (8.0–23.2)
	*p*-value	0.549	0.047	0.002	0.003	0.008	0.007
N stage	N0	−98.6 (−103.2–−92.9)	−86.1 (−93.7–−80.7)	−86.4 (−95.5–−78.9)	−72.7 (−79.0–−67.5)	13.2 (4.7–18.1)	12.8 (8.0–21.0)
	N1	−97.9 (−102.4–−91.8)	−87.4 (−94.2–−78.8)	−87.5 (−94.9–−75.6)	−74.4 (−80.9–−65.4)	10.3 (2.9–21.5)	13.2 (6.3–21.7)
	N2	−99.7 (−102.9–−94.0)	−88.4 (−94.0–−79.9)	−88.1 (−95.7–−74.2)	−74.1 (−78.5–−67.4)	9.5 (5.5–19.5)	14.4 (8.6–19.8)
	N3	−97.4 (−103.5–−89.0)	−86.6 (−92.5–−77.2)	−80.3 (−93.0–−70.3)	−70.3 (−77.8–−65.6)	16.2 (4.9–25.2)	16.9 (10.1–24.4)
	*p*-value	0.716	0.744	0.072	0.065	0.136	0.292
TNM stage	Stage II	−99.0 (−103.1–−93.3)	−87.4 (−94.6–−80.1)	−88.4 (−95.3–−80.3)	−74.4 (−80.5–−68.8)	10.0 (8.0–12.8)	12.5 (7.0–20.8)
	Stage III	−97.9 (−103.1–−89.9)	−84.8 (−91.5–−77.5)	−71.1 (−79.7–−66.6)	−70.1 (−76.7–−63.6)	15.5 (5.7–25.2)	16.4 (10.1–24.4)
	*p*-value	0.421	0.063	<0.001	<0.001	0.001	0.014

All values are median (interquartile range). CT1, pre-operative computed tomography; CT2, 6-month post-operative computed tomography; HU, Hounsfield unit; SAT, subcutaneous adipose tissue; VAT, visceral adipose tissue; ΔSAT HU, percent changes in mean computed tomography-attenuation for subcutaneous adipose tissue between pre-operative and 6-month post-operative scans; ΔVAT HU, percent changes in mean computed tomography-attenuation for visceral adipose tissue between pre-operative and 6-month post-operative scans.

**Table 3:** Univariate survival analysis of SAT and VAT HU parameters for RFS, peritoneal RFS, and OS.

Variables	RFS		Peritoneal RFS		OS	
	*p*-Value	Hazard Ratio (95% CI)	*p*-Value	Hazard Ratio (95% CI)	*p*-Value	Hazard Ratio (95% CI)
SAT HU on CT1 (<−97.2 HU vs. ≥−97.2 HU)	0.160	1.375 (0.881–2.144)	0.305	1.368 (0.752–2.489)	0.234	1.456 (0.787–2.695)
VAT HU on CT1 (<−82.9 HU vs. ≥−82.9 HU)	0.046	1.519 (1.007–2.372)	0.048	1.826 (1.004–3.322)	0.011	2.231 (1.203–4.136)
SAT HU on CT2 (<−82.3 HU vs. ≥−82.3 HU)	0.020	2.405 (1.107–3.89)	0.010	2.290 (1.222–4.290)	0.010	2.438 (1.226–4.656)
VAT HU on CT2 (<−70.0 HU vs. ≥−70.0 HU)	<0.001	3.051 (1.924–4.838)	0.001	2.892 (1.557–5.372)	<0.001	3.904 (2.017–7.557)
ΔSAT HU (<9.3% vs. ≥9.3%)	0.002	2.438 (1.388–4.283)	0.041	2.156 (1.034–4.495)	0.028	2.380 (1.099–5.156)
ΔVAT HU (<20.3% vs. ≥20.3%)	<0.001	2.369 (1.516–3.702)	0.020	2.044 (1.122–3.725)	0.016	2.130 (1.149–3.947)

CI, confidence interval; CT1, pre-operative computed tomography; CT2, 6-month post-operative computed tomography; HU, Hounsfield unit; OS, overall survival; RFS, recurrence-free survival; SAT, subcutaneous adipose tissue; VAT, visceral adipose tissue; ΔSAT HU, percent changes in mean computed tomography-attenuation for subcutaneous adipose tissue between pre-operative and 6-month post-operative scans; ΔVAT HU, percent changes in mean computed tomography-attenuation for visceral adipose tissue between pre-operative and 6-month post-operative scans.

**Table 4:** Multivariate survival analysis of SAT and VAT HU parameters for RFS, peritoneal RFS, and OS after adjusting for age, sex, histopathological classification, T stage, and N stage. ΔVAT HU was an independent predictor for RFS, peritoneal RFS, and OS, and VAT HU on CT1 and VAT HU on CT2 significantly predicted OS.

Variables	RFS		Peritoneal RFS		OS	
	*p*-Value	Hazard Ratio (95% CI)	*p*-Value	Hazard Ratio (95% CI)	*p*-Value	Hazard Ratio (95% CI)
VAT HU on CT1 (<−82.9 HU vs. ≥−82.9 HU)	0.190	1.403 (0.845–2.328)	0.072	1.797 (0.939–3.632)	0.049	2.016 (1.001–4.059)
ΔSAT HU (<9.3% vs. ≥9.3%)	0.382	1.341 (0.695–2.589)	0.753	1.146 (0.491–2.675)	0.333	1.541 (0.612–3.701)
SAT HU on CT2 (<−82.3 HU vs. ≥−82.3 HU)	0.101	1.555 (0.908–2.553)	0.401	1.331 (0.682–2.600)	0.235	1.518 (0.763–3.021)
VAT HU on CT2 (<−70.0 HU vs. ≥−70.0 HU)	0.053	2.065 (0.996–3.387)	0.066	1.992 (0.923–3.920)	0.009	2.515 (1.267–4.991)
ΔVAT HU (<20.3% vs. ≥20.3%)	0.002	2.437 (1.385–4.288)	0.023	2.457 (1.135–5.318)	0.043	2.204 (1.025–4.741)

CI, confidence interval; CT1, pre-operative computed tomography; CT2, 6-month post-operative computed tomography; HU, Hounsfield unit; OS, overall survival; RFS, recurrence-free survival; SAT, subcutaneous adipose tissue; VAT, visceral adipose tissue; ΔSAT HU, percent changes in mean computed tomography-attenuation for subcutaneous adipose tissue between pre-operative and 6-month post-operative scans; ΔVAT HU, percent changes in mean computed tomography-attenuation for visceral adipose tissue between pre-operative and 6-month post-operative scans.

## Data Availability

The datasets presented in this study are available on request from the corresponding author due to ethical restrictions.
